# Monthly Summary

**Published:** 1900-02

**Authors:** 


### Monthly Summary.

SOLID CAST GOLD CUSPS FOR CROWNS.—Some time since I was present at a jewelers when a gentleman came in with a pair of link cuff-buttons, and asked the proprietor if he could duplicate them. The jeweler replied that he would try to procure from the manufacturers the duplicate design, but that he doubted that he could purchase it, as the dies were probably laid aside for more recent designs.

The gentleman expressed his desire to have it duplicated, and the jeweler replied that if he could not secure it at the manufacturers he would make one himself. "But if I make one it will contain much more gold and be much heavier than these." "All right," replied his customer. "I am particular to replace the one I lost, as it is a keepsake."

The next evening I saw the finished ornament—a beautiful piece of work. Handing me the eyeglass, he requested me to detect the difference between the die-struck one and his duplicate of it. To the naked eye it was perfect, and the glass showed a remarkably sharp casting that was built little different from the work of the hydraulic die-press.

He then showed me the process, that struck me is of value to dentists who may need a solid cusp crown, or even to make a casting for several teeth in case of a bridge. He takes a piece of cuttlefish bone, such as sold for use in bird cages, and dresses the soft side flat with a coarse file—a rubber file will do it nicely—and presses the piece he wishes to duplicate into it to the required depth. Another piece of cuttlefish bone, filed flat, is placed over it, and a hole is gouged out in the first piece with a channel to the mold thus formed. The two pieces are then wired together, and scrap gold placed in the second hole is melted with the blowpipe; and when at proper heat the mold is filled and the cast is made.

With the excellent steel dies that we now have it is the work of but a few minutes to make a cast cusp by this method, and even a natural tooth would make a mold that would be desirable, as it would duplicate exactly every cusp and fissure.

From this suggestion it is but a step, and only a matter of detail, to cast the cusps for several teeth for a bridge using a dummy of type metal to make the mold in the cuttlefish bone.—W. H. Mitchell, D. D. S., in Dental Cosmos.

---

UNUSUAL EFFECT OF COCAINE.—Although, possibly, of no scientific importance, the following case is not without interest. It was presented to the Societe de Chirurgie, and is reported in the *Medical Press:* M. Raymond presented a young girl, aged eighteen, who was found some time ago in a profound sleep in a railway wagon. The repeated efforts of the officials to awaken her proving unsuccessful, she was carried to the Salpetriere Hospital. Here, it was only at the end of the third day that she was awakened artificially. The sleep was calm, the respiration and the pulse normal; the eyelids were closed, the face pale, and the muscular relaxation complete; in a word, the sleep was absolutely natural. When she came to herself she gave the following interesting account of her antecedents. At the beginning of the present year she went to a dentist to have a tooth pulled, and as she feared greatly the pain of the operation, the dentist employed cocaine. But after the tooth was drawn she was seized with convulsions and finally fell into a deep sleep, and for three hours the dentist tried in vain to rouse her. Carried to the hospital, she awoke the following morning. For three months all was going on well, when one day she went to sleep in the train, and, carried again to the Lariboisiere Hospital, she did not awaken until the seventh day. A few days after she was seized with sleep in the street, and was for the third time received at the hospital, where she remained asleep ten days. This time she remained under treatment three months. M. Raymond, after passing in review the possible causes of this phenomenon, said that he had fixed his opinion on hysteria, and gave the history of several other cases from a similar cause.—Dental Record.

---

DENTISTRY IN THE PHILIPPINES.—Writing from Manila an American dentist, recently established there, says: "A Dental Board has been organized here in connection with the Board of Health, and I understand that a numher of the dentists now practicing will be required to pass an examination. At the present time this is not a good location. There are a large number of dentists here, and more are arriving by each boat. Several have given it up after a short trial, and either gone farther, or else have returned to the States. There are no accommodations here for a dentist. No gas, no electric light in day time, no gasoline. One must do his soldering by the use of a native oil, or with a very poor quality of alcohol."—Items of Interest.

---

Many persons tolerate more filth and disease in their mouths than they would on their feet! The mouth is the portal of life and health!—"Dominion Dental Journal."

